# Assessment of Vegetation Indices Derived by UAV Imagery for Durum Wheat Phenotyping under a Water Limited and Heat Stressed Mediterranean Environment

**DOI:** 10.3389/fpls.2017.01114

**Published:** 2017-06-26

**Authors:** Angelos C. Kyratzis, Dimitrios P. Skarlatos, George C. Menexes, Vasileios F. Vamvakousis, Andreas Katsiotis

**Affiliations:** ^1^Department of Vegetable Crops, Agricultural Research InstituteNicosia, Cyprus; ^2^Department of Agricultural Sciences, Biotechnology and Food Science, Cyprus University of TechnologyLimassol, Cyprus; ^3^Department of Civil Engineering and Geomatics, Cyprus University of TechnologyLimassol, Cyprus; ^4^Laboratory of Agronomy, School of Agriculture, Aristotle University of ThessalonikiThessaloniki, Greece

**Keywords:** spectral vegetation indices, UAV imagery, stress, durum wheat, high-throughput phenotyping

## Abstract

There is growing interest for using Spectral Vegetation Indices (SVI) derived by Unmanned Aerial Vehicle (UAV) imagery as a fast and cost-efficient tool for plant phenotyping. The development of such tools is of paramount importance to continue progress through plant breeding, especially in the Mediterranean basin, where climate change is expected to further increase yield uncertainty. In the present study, Normalized Difference Vegetation Index (NDVI), Simple Ratio (SR) and Green Normalized Difference Vegetation Index (GNDVI) derived from UAV imagery were calculated for two consecutive years in a set of twenty durum wheat varieties grown under a water limited and heat stressed environment. Statistically significant differences between genotypes were observed for SVIs. GNDVI explained more variability than NDVI and SR, when recorded at booting. GNDVI was significantly correlated with grain yield when recorded at booting and anthesis during the 1st and 2nd year, respectively, while NDVI was correlated to grain yield when recorded at booting, but only for the 1st year. These results suggest that GNDVI has a better discriminating efficiency and can be a better predictor of yield when recorded at early reproductive stages. The predictive ability of SVIs was affected by plant phenology. Correlations of grain yield with SVIs were stronger as the correlations of SVIs with heading were weaker or not significant. NDVIs recorded at the experimental site were significantly correlated with grain yield of the same set of genotypes grown in other environments. Both positive and negative correlations were observed indicating that the environmental conditions during grain filling can affect the sign of the correlations. These findings highlight the potential use of SVIs derived by UAV imagery for durum wheat phenotyping under low yielding Mediterranean conditions.

## Introduction

Drought stress, as a combination of water deficit and high temperature, is the main constraint limiting grain yield of cereals in the Mediterranean basin (Araus et al., 2002). This geographic area is expected to face more severe drought and an increase in average temperature in the near future, due to climate change (Giorgi and Lionello, 2008), increasing yield uncertainty of rain-fed crops. Improving crop productivity in drought-prone environments is a daunting challenge. Extensive plant phenotyping and integration of cost effective technologies are considered prerequisites to achieve progress through plant improvement (Reynolds and Tuberosa, 2008). Furthermore, advances in phenotyping are likely to be essential in capitalizing developments in conventional, molecular and transgenic breeding, and ensuring genetic improvement of crops for future food security (Araus and Cairns, 2014).

Remote sensing methods hold great potential as a tool for: (a) high throughput phenotyping for plant breeding (Deery et al., 2014; Sankaran et al., 2015), (b) decision making for precision agriculture (Zhang and Kovacs, 2012; Gago et al., 2015), (c) predicting yields (Son et al., 2014), and (d) predicting spatial field variability in experimental sites (Zaman-Allah et al., 2015). Their usefulness rely on the fact that they are non-destructive, non-invasive, fast and cost-efficient, well-correlated with agronomical and important physiological crop traits (Reynolds et al., 2015).

The most common procedure to extract information about crops from remote sensing is through the estimation of Spectral Vegetation Indices (SVI), which are based on formulations fitted with the light reflected by the canopy at different wavelengths (e.g., ratios and differences). The wavelengths are within the visible and the near infrared electromagnetic spectrum. Several SVIs have been proposed and are widely used, such as the Normalized Difference Vegetation Index (NDVI), the Simple Ratio (SR) and the Green Normalized Difference Vegetation Index (GNDVI). The existence of genetic variability for SVIs was reported by several authors (Babar et al., 2006b; Prasad et al., 2007b; Gutierrez et al., 2010; Gizaw et al., 2016a). SVIs were associated with important traits of cereal crops, such as grain yield under stressed conditions (i.e., Bort et al., 2005; Lobos et al., 2014; Bowman et al., 2015; Tattaris et al., 2016; Yousfi et al., 2016). However, some authors argued that under severe stress conditions, SVIs might be less efficient because genotypes are not able to express their yield potentiality (Royo et al., 2003; Babar et al., 2006c).

The majority of previous studies were conducted with hand held sensors; however, ground measurements face several constrains (Chapman et al., 2014; Deery et al., 2014; Gago et al., 2015; Reynolds et al., 2015; Sankaran et al., 2015; Tattaris et al., 2016). Some of these constrains can be eliminated using low altitude aerial platforms. Zhang and Kovacs (2012) stated that imagery taken by low altitude aerial systems is promising, given its low cost of operation, high spatial and temporal resolution, and its flexibility in image acquisition programming. Measurements from trials can be taken when they are not accessible to ground platforms, e.g., due to water-logged or tall crops (Chapman et al., 2014). Other advantages are the limited confounded effects caused by environmental drift due to simultaneous data collection and more robust image analysis tools (Reynolds et al., 2015; Tattaris et al., 2016), wider viewing angle from the air, and absence of physical contact, hence no mechanical distraction of the growing crop (Liebisch et al., 2015). Although UAVs can carry lower payload than other aerial vectors, they enable greater flight control and autonomy (Araus and Cairns, 2014) and are less affected by the wind (Deery et al., 2014; Tattaris et al., 2014). Recent studies revealed that correlations between SVIs and agronomic traits derived from airborne imagery are similar, or even stronger, than correlations derived from ground measurements (Tattaris et al., 2014, 2016; Zaman-Allah et al., 2015; Rasmussen et al., 2016). Measurements can be taken by a wide array of different sensors including conventional digital cameras (Araus and Cairns, 2014; Sankaran et al., 2015), that have the advantage of low cost and low weight (Hunt et al., 2010), and can be easily mounted on UAVs and other aerial vectors (Ball and Konzak, 1993; Lelong et al., 2008; Liebisch et al., 2015; Rasmussen et al., 2016). The fast and cost efficient nature of UAV imagery allows multiple measurements during grain filling. Multiple measurements are necessary because the optimum recording stage is likely to vary with experiment (i.e., Bort et al., 2005; Bowman et al., 2015). The efficiency of SVIs is also affected by plant phenology, thus multiple measurements allow the calculation of parameters that are less related with phenology (Lopes and Reynolds, 2012; Montazeaud et al., 2016).

The successful implementation of such technologies relies on the characteristics of the UAV including stability, safety, control, reliability, positioning, autonomy, sensor mount, controller, sensor characteristics and image and data processing (Chapman et al., 2014; Sankaran et al., 2015). It is then necessary to assess the reliability of aerial remote sensing approaches with direct plant-derived data (Lelong et al., 2008; Gago et al., 2015; Liebisch et al., 2015). A number of studies investigated the potential use of imagery derived from sensors mounted on UAVs and other aerial vectors for plant breeding (Ball and Konzak, 1993; Hoyos-Villegas and Fritschi, 2013; Chapman et al., 2014; Liebisch et al., 2015; Zaman-Allah et al., 2015; Rutkoski et al., 2016) and precision agriculture (Lelong et al., 2008; Hunt et al., 2010; Khot et al., 2016; Rasmussen et al., 2016). Nevertheless, studies conducted under severely stressed Mediterranean conditions are very limited (Gonzalez-Dugo et al., 2015).

The main scope of the present work is to investigate the usefulness of SVIs (NDVI, SR and GNDVI) derived from UAV imagery for plant phenotyping under a water limited and heat stressed Mediterranean environment. Durum wheat, a predominant stable crop cultivated in the Mediterranean basin, was selected for this study. A fast and cost effective method to estimate SVIs by UAV mounted with digital cameras is described. Genotypic effects of SVIs and agronomic and other physiological traits are presented. Correlations between SVIs and photosynthetic pigments, SPAD measurements, grain yield and other agronomic traits are discussed.

## Materials and Methods

### Plant Material

Twenty durum wheat varieties (*Triticum turgidum* subsp. *durum*) were selected for the present study. Six varieties were bred by the Cypriot National Breeding Program and represent the main commercial varieties cultivated in Cyprus for the last 40 years. The other 14 varieties were released by other breeding programs targeting areas with similar climatic conditions (**Table 1**).

**Table 1:** List of the durum wheat varieties used in the present study.

Name	Year of release	Country of registration/Origin	Name	Year of release	Country of registration/Origin
Aronas	1977	Cyprus	Pisti	2008	Greece
Mesaoria	1982	Cyprus	Simeto	1988	Italy
Karpasia	1985	Cyprus	Duilio	1984	Italy
Macedonia	1994	Cyprus	Iride	1996	Italy
Ourania	2006	Cyprus	Claudio	1998	Italy
Hekabe	2003	Cyprus	Svevo	1996	Italy
Anna	2000	Greece	Adnan2		ICARDA^∗^
Atlas	1995	Greece	Omrabi5		ICARDA^∗^
Matt	2003	Greece	Korifla		ICARDA^∗^
Mexikali81	1985	Greece	Waha		ICARDA^∗^

*^∗^International Center for Agricultural Research in the Dry Areas.*

### Experimental Conditions and Field Design

**FIGURE 1:**
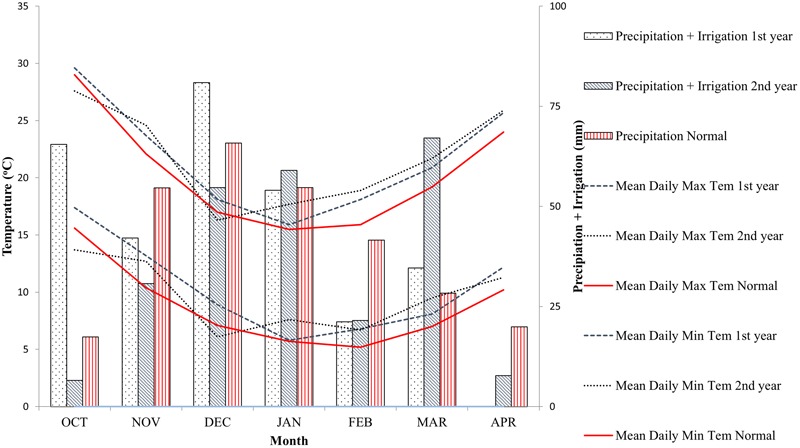
Environmental conditions during the test years and normal conditions at Athalassa experimental station.

Experiments were conducted at Athalassa experimental station (35°08′N, 33°24′E) for two consecutive growing seasons (2012/2013 – year 1 and 2013/2014 – year 2). Athalassa has shallow sandy clay loam soil and rather low precipitation during crop cycle, resulting to drought stress during heading and grain filling. In addition, the rather high day temperature in spring and the frequent occurrence of extreme high temperatures during grain filling very often result to heat stress conditions (**Figure 1**). Crop failure and complete loss of yield frequently occurs in this area.

The experimental design was a randomized complete block with four and five replications the 1st year and the 2nd year, respectively. Six row plots, 8 m long, spaced apart 0.175 m were used. Seed rate was adjusted to 226 germinating seeds m^-2^. Experiments were sown at the end of November and 60 Kg ha^-1^ of N_2_ and P_2_O_5_ were applied before sowing_._ Weeds were chemically controlled at tillering (Atlantis^®^, Bayer, Illoxan^®^, Bayer, Granstar^®^, DuPont). Additional irrigation was applied during booting (30 mm) in the 1st year, and during tillering (50 mm) and booting (50 mm) in the 2nd year. The plants received no supplementary irrigation or rainfall from heading to physiological maturity during the 1st year, while in the 2nd year received only a negligible amount of rainfall when most of the plants had reached physiological maturity.

### Measurements of Agronomic Traits

Heading date was recorded when the ears of 50% of the tillers had emerged from the flag leaf sheaths for approximately half their length and was expressed as growing degree days from emergence to heading. Physiological maturity was recorded when 50% of the spikes in the plot showed total loss of green color and was expressed as growing degree days from heading to physiological maturity. Growing degree days were calculated as described by Aparicio et al. (2000). Plant height was recorded as an average of three measurements per plot at physiological maturity, excluding awns. Number of fertile tillers per m^2^ was estimated at physiological maturity from four rows, each one 1 m long, randomly selected on the 2nd and the 5th rows. Plants from two rows, each one 1 m long, were randomly selected on the 2nd and 5th row and hand harvested to estimate the number of seeds per spike. The plots were mechanically harvested on May and grain yield was recorder at 12% moisture level. Before harvesting, the two external rows and half meter from both ends of the plots were discarded to avoid the boarding effect (Ceccareli and Grando, 1996). Thousand kernel weight was calculated as the mean weight of two samples of 200 seeds per plot and expressed in g. Volume weight was measured with a 0.5L chondrometer (Seedburo) and expressed as Kg hl^-1^.

### Measurements of Photosynthetic Pigments

Extraction of photosynthetic pigments, chlorophyll a, chlorophyll b, carotenoids, anthocyanins was carried out as described by Richardson et al. (2002), setting the extraction time to 2 h. Six disks from three flag leaves were used for the extraction from each experimental plot. The disks were sampled 5 and 10 cm apart from the base and the tip of the flag leaf, respectively. The area of each disk was 0.28 cm^2^. The concentrations of the pigments (g L^-1^) were calculated according to the equations used by Misra and Dey (2013), which are based on the data published by Lichtenthaler (1987). Sampling was done at milk stage. Chlorophyll content was also measured with a SPAD 502, Konica, Minolta during the 2nd year. Data were recorded the same dates as the UAV flights from six flag leaves randomly selected from each plot. Two measurements were taken from each leaf.

### UAV Flights, Image Acquisition and Processing

Spectral Vegetation Indices (Elvidge and Chen, 1995; Haboudane et al., 2002) were measured using autonomous UAV. Two flights were carried out during the 1st year when most varieties were at booting and milk stages. The four flights carried out during the 2nd year were performed when most varieties were at heading, anthesis, milk and dough stages.

The autonomous UAVs used for the present study were the fixed wing SwingletCam from Sensfly (1st year) and the multicopterHexa Y from 3D Robotics (2nd year). Both UAVs are fully capable for completely autonomous flight from takeoff to landing, requiring minimum expertise from the operator. Cameras used on board the SwingletCam were provided by SenseFly as part of the package. They were a Canon IXUS 220 HS for RGB photos, and a modified near infrared Canon Powershot ELPH 300 HS. Onboard the multicopter, the Canon IXUS 130 IS was used to take RGB photos and a modified near infrared Canon Powershot SX260 HS was used for near infrared photograph. Two flights were performed, one right after the other, with the exact same flight plan, but with different cameras. This method suggests that RGB and NIF photos were not taken simultaneously but with a time gap of 10 to 20 min, depending on plot dispersion. A Leica Viva dual Global Positioning System (GPS) in Real Time Kinematic (RTK) mode was used for ground control point measurements. Prior to the flight, simple white A4 sheets were laid down on the ground as control points.

Flights were conducted at varying heights from 72 up to 140 m and ground pixel sizes varying from 2.0 to 4.3 cm. The variation of ground pixel size is of no importance since the final orthophotos created, for every epoch, had 5.0 cm pixel size, larger than the ones in the original photography. Although the whole area of the crop fields could have been included in a single aerial image from the aforementioned flying height, using a 5.0 cm pixel size, was necessary to capture multiple photos in order to create a Digital Elevation Model (DEM), necessary for the orthorectification and georeferencing process.

**FIGURE 2:**
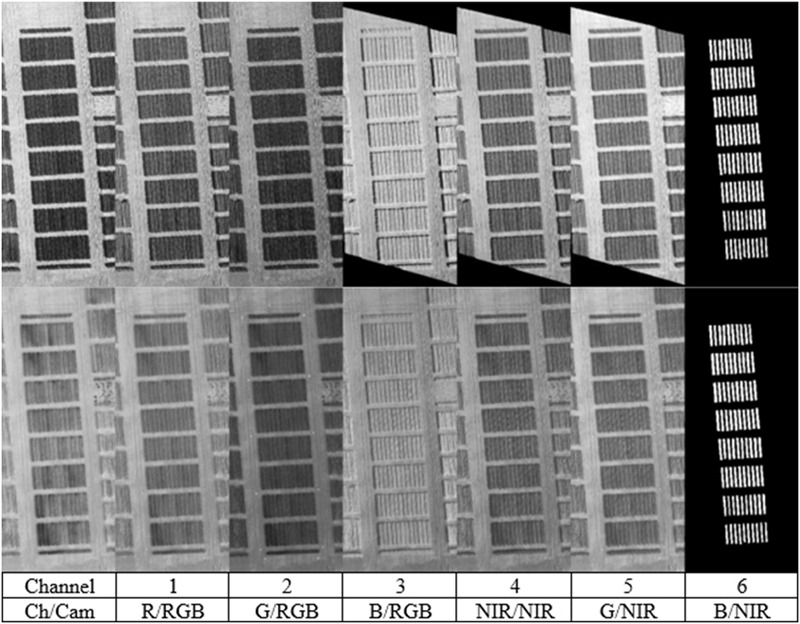
Channel integration, from the two flights (booting and milk stages) over the 1st year. The last image on the right, shows all experiments’ masks combined, similar in both cases.

All photos were processed using Agisoft’sPhotoscan (version 1.0) to produce georeferenced real color and near infrared orthophotos. During this process the original imagery is orthorectified and georeferenced to ensure that each pixel, at every epoch, of the real color and near infrared orthophotos correspond perfectly to each other. As a byproduct of the process, a DEM of the ground and the canopy of the crop surface was produced. Slight color differentiation is likely to happen, even among photos, from the same camera because of light conditions, camera settings, sun reflection and camera angles. Mosaicking of photos during the last phase of orthophoto mosaicking process, produces misalignments and color shifting due to automatic software color matching and correction. In order to avoid the color changes, a single photo from each set (true color or near infrared), covering the crop area was selected to create the final orthophotos. Orthophotos were created with user specified coordinates values, as to ensure full correspondence over overlapping pixels. Final orthophotos had the exact same number of pixels and three channels each (**Figure 2** and Supplementary Table S1 for the position of individual varieties in **Figure 2**). After the creation of the true color and near infrared orthophotos, they were loaded into Matlab software (version 12) for further processing. Within Matlab they were stacked to form a six channel photo, according to **Figure 2**. With arithmetical functions among the pixels, several SVIs were calculated. Once the multispectral orthophotos were created, masks over each crop were manually created. The masks were concentrated over the crop’s main body, excluding the crop’s edges where mixing with the next variation might have caused misleading results. Nevertheless, even by reducing extend and pixels of each plot, an average of 9350 pixels were left per plot. Based on the manually collected masks (**Figure 2**, on the far right), they were combined in each experimental plot. The following indices (Agapiou et al., 2012) were calculated for each experimental plot:

$$\begin{array}{c} \text{NDVI}=\left(R_{\text{NIR}}-R_{\text{red}}\right)/\left(R_{\text{NIR}}+R_{\text{red}}\right)\;\\ \text{GNDVI}=\left(R_{\text{NIR}}-R_{\text{green}}\right)/\left(R_{\text{NIR}}+R_{\text{green}}\right)\\ \text{SR}=R_{\text{NIR}}/R_{\text{red}}\; \end{array}$$

The 2nd year, GNDVI was calculated only for the first two flights, i.e., at heading and booting.

### Statistical Analysis

Combined analysis over years was conducted for agronomic traits considering genotypes and years as fixed factors. One-way ANOVA was conducted for agronomic traits, SVIs, SPAD measurements and photosynthetic pigments for each growth stage and year. ANOVA was also conducted for SVIs and SPAD measurements considering all the growth stages together during each year. Pearson correlation coefficients on genotype means were estimated between agronomic traits, photosynthetic pigments, SPAD measurements and SVIs. Correlations between NDVIs, SPAD values, and chlorophyll b recorded at Athalassa with grain yield recorded in experiments with the same set of genotypes grown at different locations (Achelia and Dromolaxia experimental stations, Cyprus) are also presented. Principal Component Analysis was performed using the direct oblimin rotation method to explore relationships among variables. The PCs with eigenvalues greater than 1 were selected and coefficients greater than 0.3 are shown. Stepwise regression analysis was performed on genotype means to investigate SVIs, SPAD measurements and photosynthetic pigments contribution to grain yield. All analyses were carried out using SPSS (IBM, SPSS ver 22).

## Results

### Genotypic Effects

Analysis of Variance *F*-values for different traits among genotypes including means, maximum and minimum values, and the Coefficient of Variations (CVs) for the 2 years and the combined analysis over years for the agronomic traits are shown in **Table 2**. There was genetic variability between genotypes, except for grain yield in the 2nd year and for the combined analysis over years. Environmental conditions affected seeds per spike, volume weight, thousand kernel weight and growing degree days to heading. The interactions were weaker or non-significant. Statistically significant differences between genotypes were also observed for SVIs, except for SR at milk stage the 2nd year, SPAD values at all stages and for photosynthetic pigments (**Table 3**). The SVIs differences were more profound during the 1st year. NDVI and SR explained more variation at milk stage, contrary to GNDVI, which explained more variation at booting stage during the 1st year.

**Table 2:** Analysis of Variance *F*-values for genotypes, Coefficients of Variation (CV), means, minimum (Min) and maximum (Max) values of grain yield (GRYLD), number of tillers per m^2^ (NTLSM), seeds per spike (SPS), volume weight (VW), thousand kernel weight (TKW), plant height (PH), growing degree days to heading (GDDHD) and growing degree days from heading to physiological maturity (GDDPM).

	1st year							
	GRYLD (Kg/ha)	NTLSM	SPS	VW (Kg/hl)	TKW (g)	PH (cm)	GDDHD	GDDPM
F genotype	2.443^∗∗^	4.371^∗∗∗^	15.814^∗∗∗^	13.973^∗∗∗^	20.537^∗∗∗^	5.202^∗∗∗^	50.912^∗∗∗^	–
CV	16.59	14.55	8.20	2.11	6.39	5.10	1.13	–
Mean	1723	252.71	27.60	66.55	23.56	78.93	1275	–
Min	1247	206.79	22.42	62.35	19.24	68.88	1203	–
Max	2126	337.50	38.81	71.05	33.50	87.25	1398	–

	**2nd year**							
F genotype	1.162	2.772^∗∗^	14.051^∗∗∗^	5.709^∗∗∗^	4.010^∗∗∗^	5.111^∗∗∗^	23.542^∗∗∗^	3.128^∗∗∗^
CV	25.72	15.00	11.42	2.87	12.19	5.30	1.33	7.47
Mean	1651	258.49	24.00	70.34	27.03	81.30	1360	558.40
Min	995	172.38	18.13	65	21.29	70.73	1296	443.58
Max	2013	312.50	36.53	75	35.35	89.80	1437	606.33

	**Combined analysis**							
F genotype	1.260	4.679^∗∗∗^	25.267^∗∗∗^	11.461^∗∗∗^	8.868^∗∗∗^	6.751^∗∗∗^	43.288^∗∗∗^	–
F year	0.017	0.288	50.007^∗∗∗^	78.109^∗∗∗^	13.216^∗∗∗^	0.019	540.147^∗∗^	–
F genotype x year	1.768^∗^	1.919^∗^	2.717^∗∗∗^	1.754^∗^	1.307	1.657	2.344^∗∗^	–
Mean	1685	255.79	25.69	68.57	25.41	80.19	1320	–
CV	21.82	15.62	9.91	2.89	11.70	5.71	1.52	–

*^∗^*p* < 0.05, ^∗∗^*p* < 0.01, ^∗∗∗^*p* < 0.001.*

**Table 3:** Analysis of Variance *F*-values for genotypes, Coefficients of Variation (CV), means, minimum (Min) and maximum (Max) values for SVIs, SPAD values and photosynthetic pigments.

	1st year	2nd year		1st year	2nd year		2nd year	
	**NDVI booting**	**NDVI heading**	**NDVI anthesis**	**NDVI milk**	**NDVI milk**	**NDVI dough**	**SPAD heading**	**SPAD anthesis**

F genotype	8.086^∗∗∗^	2.464^∗∗^	2.245^∗∗^	9.937^∗∗∗^	2.042^∗^	2.261^∗∗^	9.036^∗∗∗^	5.447^∗∗∗^
CV	4.00	8.39	12.32	27.32	14.72	8.54	3.02	3.26
Mean	0.49536	0.50460	0.41274	0.11633	0.30512	0.28772	55.89	56.42
Min	0.43407	0.44609	0.34481	0.04668	0.23476	0.25044	51.68	51.94
Max	0.55610	0.56472	0.50076	0.25669	0.38853	0.34017	59.82	59.06

	**SR booting**	**SR heading**	**SR anthesis**	**SR milk**	**SR milk**	**SR dough**	**SPAD milk**	**SPAD dough**

F genotype	6.009^∗∗∗^	3.036^∗∗∗^	2.171^∗^	8.417^∗∗∗^	1.659	2.083^∗^	5.297^∗∗∗^	4.144^∗∗∗^
CV	6.46	10.25	13.41	7.46	12.24	6.02	14.44	37.07
Mean	3.09	3.16	2.54	1.27	1.95	1.82	45.86	25.96
Min	2.61	2.67	2.12	1.10	1.63	1.67	28.69	10.59
Max	3.68	3.67	3.11	1.70	2.33	2.04	59.51	49.86

	**1st year**							
	**Anthocyanin**	**Chlorophyll**	**Chlorophyll**	**Carotenoids**	**Total chlorophyll**	**GNDVI**	**GNDVI**	
	**(g/l)**	**b (g/l)**	**a (g/l)**	**(g/l)**	**(g/l)**	**booting**	**milk**	

F genotype	6.001^∗∗∗^	13.956^∗∗∗^	14.118^∗∗∗^	11.084^∗∗∗^	14.208^∗∗∗^	15.346^∗∗∗^	9.942^∗∗∗^	
CV	10.40	19.61	17.47	13.65	17.62	2.72	4.92	
Mean	0.004234	0.001045	0.008761	0.004207	0.009806	0.26742	0.21992	
Min	0.003419	0.000385	0.003651	0.002515	0.004036	0.24178	0.18053	
Max	0.005427	0.001770	0.014283	0.006068	0.016053	0.29556	0.25394	

	**2nd year**							
	**Anthocyanin**	**Chlorophyll**	**Chlorophyll**	**Carotenoids**	**Total chlorophyll**	**GNDVI**	**GNDVI**	
	**(g/l)**	**b (g/l)**	**a (g/l)**	**(g/l)**	**(g/l)**	**heading**	**anthesis**	

F genotype	2.293^∗∗^	4.318^∗∗∗^	4.945^∗∗∗^	3.492^∗∗∗^	4.885^∗∗∗^	2.440^∗∗^	2.078^∗^	
CV	15.30	27.64	24.46	20.94	24.77	8.36	10.67	
Mean	0.003599	0.001121	0.007922	0.002952	0.009044	0.23066	0.21854	
Min	0.002999	0.000721	0.005149	0.002026	0.005871	0.19939	0.18468	
Max	0.004529	0.001934	0.013303	0.004187	0.015237	0.25480	0.24731	

*^∗^*p* < 0.05, ^∗∗^*p* < 0.01, ^∗∗∗^*p* < 0.001.*

Normalized Difference Vegetation Indices and SR means were lower during the 1st year, particularly at milk stage. Since different digital cameras were used during the 2nd year, there is variation among various digital cameras due to the different sensor sensitivity at various spectral reflectances and the different lens filters used. For example, Li et al. (2010) found close relations between vegetation indices derived from three different digital cameras and canopy cover, however, the magnitude of the estimated canopy cover varied with camera. Thus, the results of the two years were analyzed independently.

There were significant differences between genotypes and growth stages when measurements from all stages were analyzed together for SVIs and for SPAD (**Table 4**). Both SVIs and SPAD values were progressively reduced as plants were reaching maturity. However, differences between milk and dough stages were not significant for NDVI and SR in the 2nd year. Furthermore, SPAD values at heading and anthesis did not differ significantly. Significant interactions between genotypes and growth stages were observed for NDVIs and GNDVIs the 1st year and for SPAD values the 2nd year.

**Table 4:** Analysis of Variance *F*-values for genotypes, growth stage, genotype × growth stage, Coefficient of Variation (CV) and means of NDVI, SR, GNDVI and SPAD values.

	1st year			2nd year			
	NDVI	SR	GNDVI	NDVI	SR	GNDVI	SPAD
F genotype	9.012^∗∗∗^	3.363^∗∗∗^	9.224^∗∗∗^	4.866^∗∗∗^	4.434^∗∗∗^	3.282^∗∗∗^	4.937^∗∗∗^
F growth stage	5302.67^∗∗∗^	2011.58^∗∗∗^	494.78^∗∗∗^	316.69^∗∗∗^	267.18^∗∗∗^	11.91^∗∗^	240.90^∗∗∗^
F genotype x stage	3.324^∗∗∗^	1.425	1.775^∗^	0.263	0.433	0.222	1.514^∗^
CV	10.69	11.67	5.51	14.02	14.74	10.92	18.91
Mean	0.30585	2.18	0.24367	0.37755	2.37	0.22460	46.03

*^∗^*p* < 0.05, ^∗∗^*p* < 0.01, ^∗∗∗^*p* < 0.001.*

### Associations between SVI Indices and Photosynthetic Pigments

Correlations between SVIs, SPAD values and photosynthetic pigments during the 1st year and the 2nd year, respectively, are shown at Supplementary Tables S2, S3. There were very strong correlations between SVIs recorded at each growth stage and at different growth stages. SPAD values at milk stage were highly correlated with SPAD values at dough stage. Weaker, although significant correlations were also observed between SPAD values at heading and SPAD values at anthesis and at milk stage. SPAD values at milk and dough stages showed significant correlations with SVIs. The correlations were stronger at dough stage. There were significant correlations between chlorophyll pigments and carotenoids with SVIs with the exception of GNDVI at heading for the 2nd year. Anthocyanin correlations were non-significant or were weak. SPAD values were significantly correlated with chlorophyll pigments and carotenoids, except from SPAD at anthesis.

There were significant correlations between SVIs recorded at milk stage the 1st year with SVIs, SPAD values at milk and dough stages and photosynthetic pigments recorded the 2nd year (Supplementary Table S4). SVIs recorded at booting the 1st year were significantly correlated only with chlorophyll pigments, carotenoids and SPAD values at heading, anthesis and milk stages.

### Associations with Agronomic Traits

Significant correlations were obtained between grain yield with NDVIs and GNDVIs at booting the 1st year and with GNDVIs at anthesis the 2nd year (**Table 5**). The correlations were higher the 1st year, when genetic variation in grain yield was also significant. According to stepwise regression results, GNDVI at booting and at anthesis explained 31.8 and 21.5% of grain yield variability for the 1st year and the 2nd year, respectively. Standardized beta coefficients were positive in both cases (**Table 6**).

**Table 5:** Pearson correlations between SVIs and grain yield at different growth stages.

Year	Growth stage	Correlations
1st year	NDVI booting	0.526^∗^
	GNDVI booting	0.564^∗∗^
	SR booting	0.461
	NDVI milk stage	0.418
	GNDVI milk stage	0.419
	SR milk stage	0.384
2nd year	NDVI heading	0.426
	GNDVI heading	0.318
	SR heading	0.410
	NDVI anthesis	0.438
	GNDVI anthesis	0.464^∗^
	SR anthesis	0.413
	NDVI milk stage	0.402
	SR milk stage	0.382
	NDVI dough stage	0.361
	SR dough stage	0.346

*^∗^*p* < 0.05, ^∗∗^*p* < 0.01, *n* = 20.*

**Table 6:** Stepwise regression between grain yield and anthocyanin, carotenoids, chlorophyll b, NDVI and GNDVI at booting and milk stage for the 1st year and anthocyanin, carotenoids, chlorophyll b, SPAD at milk and dough stages, NDVI at heading and milk stage and GNDVI at heading and anthesis for the 2nd year.

	Variable	Model	Standardized	*F*	
Year	enter	*R*^2^	Beta	Change	Probability
1st year	GNDVI booting	0.318	0.564	8.384	0.010
2nd year	GNDVI anthesis	0.215	0.464	4.938	0.039

The correlations between NDVIs at different growth stages, SPAD values at milk and dough stages, and chlorophyll b with grain yield, from the same set of genotypes grown in different years and locations, are presented in **Table 7**. Negative correlations were obtained between NDVI and grain yield in Dromolaxia for two consecutive years. Negative correlations between NDVI and grain yield were also observed in Achelia for one year while for the other year, NDVI was positively correlated. The best recording stage varied with experiment. Significant negative correlations were also obtained between SPAD values and chlorophyll b with grain yield, although in most cases were weaker than the NDVI.

**Table 7:** Pearson correlations between NDVI at different stages, SPAD at milk and dough stages and chlorophyll b with grain yield from the same set of genotypes grown at different years and locations (DR12, Dromolaxia 2011/12; AX12, Achelia 2011/12; ATH13, Athalassa 2012/2013; DR13, Dromolaxia 2012/13; AX13, Achelia 2012/13; ATH14, Athalassa 2013/2014).

	DR 12	AX12	ATH13	DR13	AX13	ATH14
NDVI booting ATH13	ns	0.557^∗^	–	ns	ns	ns
NDVI milk ATH13	ns	ns	–	-0.450^∗^	-0.478^∗^	ns
Chlorophyll b ATH13	ns	ns	–	ns	-0.491^∗^	ns
NDVI heading ATH14	-0.615^∗∗^	ns	ns	-0.536^∗^	ns	–
NDVI anthesis ATH14	-0.547^∗^	ns	ns	–0.574^∗∗^	ns	–
NDVI milk ATH14	-0.537^∗^	ns	ns	-0.653^∗∗^	ns	–
NDVI dough ATH14	-0454^∗^	ns	ns	-0.660^∗∗^	-0.449^∗^	–
SPAD milk ATH14	ns	ns	ns	ns	-0.613^∗∗^	–
SPAD dough ATH14	ns	ns	ns	-0.543^∗^	-0.641^∗∗^	–
Chlorophyll b ATH14	ns	ns	ns	-0.569^∗∗^	-0.566^∗∗^	–

*ns (not significant), ^∗^*p* < 0.05, ^∗∗^*p* < 0.01, *n* = 20.*

Principal Component Analysis was conducted to investigate the combinations of traits that best explained the variability. The first three PCs explained 78.46 and 79.97% of the total variance during the 1st and 2nd years, respectively (**Table 8**). For both years, the PC1 was strongly and positively associated with chlorophyll b, carotenoids, anthocyanin, SVIs at milk stage, growing degree days to heading and volume weight. The associations of SVIs recorded at earlier stages with PC1 were weaker, especially the 2nd year. Grain yield was positively related to PC1 the 1st year and to PC2 for both years. Strong and positive associations with PC2 were also observed for number of tillers per m^2^ for both years and weaker for SVIs at booting, heading and anthesis, and plant height. Growing degree days to heading were negatively related to PC2 the 1st year. Growing degree days from heading to physiological maturity were positively related to PC2 the 2nd year.

**Table 8:** Pattern matrix of the PCA analysis.

Pattern matrix							
**1st year**				**2nd year**			
	PC1	PC2	PC3		PC1	PC2	PC3

Chlorophyll b	0.963			Carotenoids	0.938		
Carotenoids	0.952			Chlorophyll b	0.933		
NDVI milk	0.921			Anthocyanin	0.887		
GNDVI milk	0.895			VW	0.837		
Anthocyanin	0.861		0.313	SPAD milk	0.784		
GDDHD	0.781	-0.389		NDVI milk	0.625	0.360	-0.356
VW	0.778		0.383	NTLSM		0.924	
GNDVI booting	0.716	0.370	-0.382	GRYLD		0.849	
NDVI booting	0.657	0.478		PH		0.764	
NTLSM		0.917	0.322	GDDPM	0.560	0.679	
GRYLD	0.417	0.645		GNDVI heading		0.332	-0.784
PH	-0.377	0.452		TKW	0.520		0.765
SPS			-0.888	GDDHD	0.561		-0.699
TKW	0.491		0.682	NDVI heading	0.336	0.416	-0.623
				GNDVI anthesis	0.361	0.473	-0.586
				SPS			-0.584
Cumulative variance (%)	50.01	66.52	78.46	Cumulative variance (%)	46.56	66.33	79.97

*PCA was based on agronomic traits, anthocyanin, carotenoids, chlorophyll b, NDVI and GNDVI at booting and milk stage for the 1st year and on the agronomic traits, anthocyanin, carotenoids, chlorophyll b, SPAD at milk stage, NDVI at heading and milk stage and GNDVI at heading and anthesis for the 2nd year. GRYLD, Grain yield; NTLSM, number of tillers per m^*2*^; SPS, seeds per spike; VW, volume weight; TKW, thousand kernel weight; PH, plant height; GDDHD, growing degree days to heading; GDDPM, growing degree days from heading to physiological maturity.*

### Implications with Phenology

**FIGURE 3:**
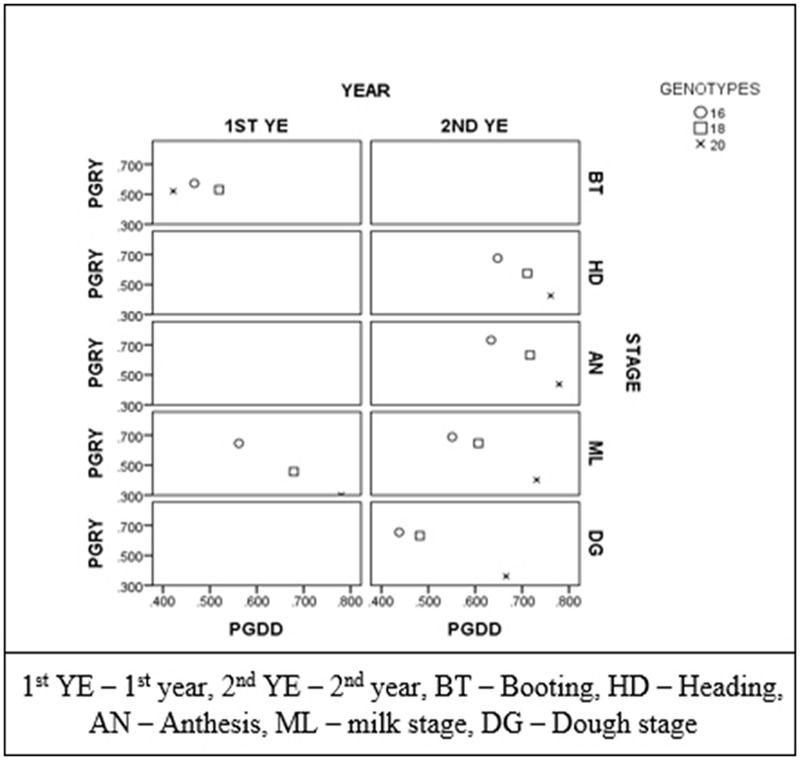
Correlation coefficients between growing degree days to heading with NDVI (PGDD) plotted against coefficients between grain yield and NDVI (PGRY) for each year and recording stage. Each point represents the correlations when all genotypes were taken into account (*n* = 20), when the two late heading genotypes were excluded (*n* = 18) and when the four late heading genotypes were excluded (*n* = 16).

In order to examine the implications of plant phenology in the ability of NDVI to predict yield, Pearson correlations between grain yield and NDVI were plotted against the correlations between growing degree days to heading and NDVI for each year and recording stage (**Figure 3**). Each point represents the correlations when all genotypes were taken into account, and when the two and four late heading genotypes were excluded. There were significant correlations between NDVI measurements and grain yield at all growth stages and years when the two and the four late heading genotypes were excluded. The correlations between grain yield and NDVI were stronger when the correlations between NDVI with growing degree days to heading were weaker.

## Discussion

There is growing interest for using SVIs derived by UAV imagery as a fast and cost efficient tool for plant phenotyping. The development of such tools is of paramount importance to continue progress through plant breeding, especially in drought prone and heat stressed environments where climate change is expected to increase yield uncertainty. Studies conducted under Mediterranean environment are limited and the intent of the present study is to elucidate the usefulness of such tools under these harsh environmental conditions.

### Genotypic Effects

Grain yields were similar (Aparicio et al., 2000; Gutierrez et al., 2010; Lobos et al., 2014) or lower from average yields reported in experiments under stressed conditions for rainfed cereal crops (Babar et al., 2006c; Lopes and Reynolds, 2012; Bowman et al., 2015; Gizaw et al., 2016a) indicating the severe stress that plants experience during their growing cycle.

The significant differences between genotypes for SVIs are in line with previous reports (i.e., Aparicio et al., 2000; Babar et al., 2006c; Prasad et al., 2007b; Gutierrez et al., 2010). The differences in the present study were more profound during the 1st year. Regarding the 2nd year, the discriminating ability of SVIs was affected by the higher experimental error due to the unusual drought conditions during the vegetative stage. Soil heterogeneity becomes more apparent under drought conditions (Masuka et al., 2012) increasing the experimental error and undermining field screenings, including phenotyping with SVIs (Zaman-Allah et al., 2015).

During the 1st year, there was less variation among genotypes for NDVI and SR at booting. This is in agreement with the findings of Royo et al. (2003), Babar et al. (2006b), and Prasad et al. (2007a). The maximum Leaf Area Index (LAI) for wheat grown under Mediterranean conditions occurs at booting. The usefulness of SR and NDVI for estimating grain yield and other important agronomic traits is limited to LAI values lower than 3 to 4 (Aparicio et al., 2000, 2002). Contrary, GNDVI explained more variation at booting during the 1st year indicating that it is less affected by high LAI values. Gitelson et al. (2002) reported that *R*_red_ sensitivity was at least three times lower than *R*_green_ when vegetation fraction was more than 60%, thus vegetation indices using green wavelength are likely to perform better at high LAI values.

The variability explained by the growth stage was much higher than the variation explained by genotypes for SVIs and SPAD values, as deduced in **Table 4**. These findings are in agreement with the results of Aparicio et al. (2002), Bort et al. (2005), Babar et al. (2006b,c), and Prasad et al. (2007b). SVI mean values progressively reduced from booting to dough stage as was shown in previous studies (i.e., Babar et al., 2006a; Prasad et al., 2007a; Gizaw et al., 2016b). The non-significant reduction from milk to dough stage in this study is justified by the fact that severe leaf senescence was present when plants were at milk stage.

Previous studies reported significant interactions between genotypes and growth stages under irrigated and stressed conditions (Babar et al., 2006b,c; Prasad et al., 2007b; Gutierrez et al., 2010; Gizaw et al., 2016a). Those authors pointed out that the interactions of growth stages and indices indicate that care must be taken to identify a suitable growth stage at which the indices will be applied to discriminate most effectively among genotypes in breeding trials. In the present study, the high correlation between SVIs recorded at different stages in the 2nd year is consistent with the non-significant interactions between growth stage and genotypes. During the 1st year, the correlations between SVIs recorded at booting and milk stage were weaker, justifying the existence of significant interactions. The interactions observed during the 1st year can be attributed to the noise induced to the data from the 1st recording stage at booting, when LAI values were at maximum. SR is less affected by the saturation effect of LAI greater than 3 compared with NDVI (Serrano et al., 2000; Aparicio et al., 2002) which might explain the lack of significant interactions for SR. Aparicio et al. (2002) reported significant interactions between genotypes and recording stage for NDVI but not for SR. Montazeaud et al. (2016) stated that NDVI saturation is not easily attained in the rainfed conditions of low yielding environments. During the 2nd year, measurements were taken at heading and onward, when NDVI saturation effect becomes less significant, reducing the noise in the data. These results indicate that, under severe stress, and for SVI measurements taken after heading, the interactions between growth stage and genotypes are likely to be low or non-significant.

### Associations between SVI Indices and Photosynthetic Pigments

Several authors stressed the strong relationship between SVIs (i.e., Bort et al., 2005; Gizaw et al., 2016b). Previous studies reported associations between SVIs recorded at different growth stages in the same environment and between SVIs recorded at different environments under more favorable (Babar et al., 2006b; Prasad et al., 2007b) and stressed conditions (Babar et al., 2006c). The results of this study are in agreement with the previously mentioned observations.

The strong positive correlations between SVIs with SPAD values and photosynthetic pigments confirm the close associations between SVIs and canopy greenness. Serrano et al. (2000) also observed significant correlations between NDVI/SR and chlorophyll a. Non-significant or very weak correlations were observed between SVIs and SPAD values at heading and anthesis, contrary to the significant positive correlations at milk and dough stages, due to SPAD values above 50, which are less reliable (Minolta SPAD502 plus manual^1^). This is further justified by the weak or non-significant correlations between SPAD values at heading and anthesis with the values recorded at milk and dough stages. Previous studies found non-significant or very weak correlations between SPAD and NDVI measurements for bread and durum wheat under Mediterranean conditions (Yousfi et al., 2016) or negative correlations for maize (Liebisch et al., 2015). Contrary, in the present study, positive significant correlations were found between SPAD values with SVIs at both milk and dough stages. This is in line with the significant positive correlations between SPAD values and photosynthetic pigments. Similarly, Babar et al. (2006a) found positive correlations between reflectance spectral indices (RARS), which are associated with photosynthetic pigments and SPAD measurements.

### Associations with Agronomic Traits

The significant correlations between grain yield and SVIs are in agreement with previous studies proposing SVIs as a mean for estimating important traits such as grain yield under heat and/or drought conditions (Aparicio et al., 2000; Bort et al., 2005; Gutierrez et al., 2010; Lobos et al., 2014; Tattaris et al., 2014, 2016; Bowman et al., 2015; Zaman-Allah et al., 2015; Gizaw et al., 2016a,b). The weak or non-significant correlations of grain yield with SPAD values and photosynthetic pigments confirm the superiority of SVIs compared to SPAD measurements as predictors of grain yield under stressed conditions (Lopes and Reynolds, 2012; Yousfi et al., 2016).

Other authors postulated that SVIs are likely to be more successful under moderate rather than under severe stressed conditions (Babar et al., 2006c), where genotypes are able to express their yield potential (Royo et al., 2003). These studies were conducted with hand-held sensors. Gonzalez-Dugo et al. (2015) reported non-significant correlations between grain yield and vegetation indices under Mediterranean conditions, derived by hyper spectral camera mounted on manned aircraft. In their study, there was only one sampling date during the critical period of grain filling. Our results indicate that SVIs derived by UAV imagery are likely to be useful in severe stressed Mediterranean conditions, with average grain yield as low as 1700 Kg/ha. In drought stressed conditions, small variability in soil depth and texture have increasingly large effects on variability (Ceccareli and Grando, 1996), thus whole plot measurements derived by UAV imagery are likely to be more representative than hand-held measurements. This also justifies the higher correlations between SVIs and grain yield compared with SPAD values and photosynthetic pigments. Multiple sampling dates are necessary from booting to physiological maturity since significant correlations with grain yield might be obtained only in one growth stage that can vary with experiment. The need for multiple measurements during the crop cycle was already stressed by several authors. For example, repeated measurements on the same genotypes over different growth stages accumulate information on the respective health of genotypes through time, thus average values across growth stages can give better predictions of yield (i.e., Babar et al., 2006c; Prasad et al., 2007b; Gizaw et al., 2016b).

An association between indices measured in one site and the yield of the same genotypes in another site, would mean that the indices could be used to predict yield in diverse environments. Significant correlations between indices measured in one environment and yield measured in a different environment were previously reported (Bort et al., 2005; Gutierrez et al., 2010; Gizaw et al., 2016a). In the present study, both positive and negative significant correlations were observed between NDVI and grain yield for the same set of genotypes grown at different environments (**Table 7**). Negative correlations between NDVI and grain yield were observed when negative correlations between grain yield and growing degree days to heading were recorded. Positive correlations were observed when the correlations between grain yield and growing degree days to heading were non-significant (data not shown). The majority of the previous studies reported positive relations between grain yield and vegetation indices. For example, Lobos et al. (2014) and Gizaw et al. (2016b) reported positive correlations between NDVI and grain yield under sever water stress, and non-significant correlations between grain yield and days to heading. However, negative correlations were reported under severe stress conditions, where negative correlations between SVIs and grain yield coexisted with negative correlations between days to heading and grain yield (Lopes et al., 2014; Rutkoski et al., 2016). Early maturing genotypes are likely to be more productive in stressed environments (Bort et al., 2005). The superiority of early maturing genotypes in their study justified the negative associations between NDVI and SR at the latest recording stage. They concluded that the changes in the values and the signs of the correlations between grain yield and reflectance indices reflect genotypic differences in response to high temperature and drought during late grain filling. The results of the present study are in agreement with their findings.

The variation explained by the first three PCA components was similar to the variation recently reported by Gizaw et al. (2016b). The first component was highly correlated with SVIs and volume weight for both years. Other studies showed less consistent correlations between SVIs and volume weight. For example, Arguello et al. (2016) reported volume weight and NDVI in the same clustering of a PCA analysis conducted under water logged conditions, but not under normal conditions. Gizaw et al. (2016b) did not find any close association between volume weight and vegetation indices. In the present study, the consistent correlations between SVIs and volume weight can be attributed to the fact that late heading genotypes had higher volume and higher SVI values. Number of tillers per m^2^, plant height, thousand kernel weight and number of seeds per spike, were less correlated to SVIs. Previous studies also reported lower and inconsistent relations between spectral reflectance indices and the above mentioned agronomic traits (Aparicio et al., 2002; Babar et al., 2006b; Lobos et al., 2014; Gizaw et al., 2016b).

### Implications with Phenology

The implications between plant phenology and SVIs can affect the correlations between SVIs and agronomic traits, particularly grain yield (Lopes and Reynolds, 2012; Tattaris et al., 2016). Principal component analysis showed a consistent strong correlation between SVIs and growing degree days to heading, as has been previously reported (Lopes et al., 2014; Lobos et al., 2014; Elazab et al., 2015; Gizaw et al., 2016b). Plant phenology affected the ability of SVI to predict yield, as it is deduced by the negative trend between correlations of NDVI with grain yield and correlations between NDVI with growing degree days to heading.

Rate of senesce, estimated as the slope of the NDVI decay against thermal time, and stay green, as an estimation of NDVI at physiological maturity, can give an independent measurement of stay green without the confounding effect of phenology (Lopes and Reynolds, 2012). In the present study, the rate of senescence and stay green were not related with grain yield (data not shown). On the contrary, NDVI values at the intercept of the slope with the *Y* axis, which estimates NDVI values at the end of booting-beginning of heading, were significantly and positively correlated with grain yield (*r* = 0.583, *p* < 0.01). The correlations were similar when the four late heading genotypes were excluded (*r* = 0.545, *p* < 0.05). The intercept NDVI was not correlated with growing degree days to heading implying that it is not related with phenology. Montazeaud et al. (2016) reported positive correlations between grain yield and maximum greenness, as estimated by NDVI measurements. Maximum greenness coexists with booting, when water is relatively available and the temperatures are still not high.

In stressed environments, biomass accumulation before heading is associated with grain yield as it is related to carbohydrate remobilization to grain during the grain filling stage (Villegas et al., 2001). Significant correlations between SVIs and biomass have been reported (Aparicio et al., 2002; Babar et al., 2006a). The positive correlation with grain yield that was observed for GNDVI recorded at booting the 1st year, and for intercept NDVI at the 2nd year might be associated with genotypes that manage to accumulate high biomass before heading. The SVI measurements at this stage were independent of phenology as it is shown by the non-significant correlations with days to heading. SVI measurements at later stages were depended from phenology and they were not associated with grain yield because late heading genotypes had higher SVI values, but they were less productive. Lopes et al. (2014) confirmed that NDVI measurements after booting are related to plant greenness and selecting for high NDVI after booting, late flowering genotypes will be selected which are low yielding. When late heading genotypes were excluded, high positive correlations were obtained implying that within a narrower range of heading, stay-green genotypes were more productive. In environments where days to heading were negatively associated with grain yield, significant negative correlations were obtained between NDVIs and grain yield. SVIs are predictors of canopy greenness (Aparicio et al., 2000), thus early maturing genotypes were associated with low SVI values.

The results of the present work highlight the potential use of SVIs derived by UAV imagery for durum wheat phenotyping under low yielding Mediterranean conditions. The optimum recording stage varied with experiment. The ability of SVIs as yield predictors was affected by plant phenology. The implications between plant phenology and SVIs derived by UAV imagery should be investigated in future studies, employing parameters that are less related to plant phenology. Other indices, such as water indices (Babar et al., 2006c; Gutierrez et al., 2010) and/or RGB indices (Elazab et al., 2015; Vergara-Diaz et al., 2016) were found to be superior compared to SVIs in field phenotyping. Additional research should be conducted in the future, addressing the performance of these indices derived from UAV imagery.

## Author Contributions

ACK and AK carried out the design of the experiment. ACK conducted the field and laboratory measurements. DS and VV carried out the UAV flights and processed the aerial imageries. GM contributed to the data analysis. ACK analyzed the data and wrote the paper under the supervision of AK and with contributions from all the other authors.

## Conflict of Interest Statement

The authors declare that the research was conducted in the absence of any commercial or financial relationships that could be construed as a potential conflict of interest.

## Footnotes

1 https://www.konicaminolta.com/instruments/download/catalog/color/pdf/spad502plus_catalog_eng.pdf
