# Supplementary material for: Assessment of Vegetation Indices Derived by UAV Imagery for Durum Wheat Phenotyping under a Water Limited and Heat Stressed Mediterranean Environment
Source: Front Plant Sci. 2017 Jun 26;8:1114. doi: 10.3389/fpls.2017.01114 (PMC5483459; doi:10.3389/fpls.2017.01114)
Supplement: Supplementary file 1 [file Table_1.docx]

**Supplementary Table 1:** Position of individual varieties in figure 2

| Guard row | | | | | | | | | | | |  |
| --- | --- | --- | --- | --- | --- | --- | --- | --- | --- | --- | --- | --- |
|  |  |  |  |  |  |  |  |  |  |  |  |  |
| Guard row | Ourania | Adnan2 | Waha | Iride | Matt | Claudio | Aronas | Hekabe | Mesaoria | Svevo | Guard row | Replication IV |
|  |  |  |  |  |  |  |  |  |  |  |  |  |
| Guard row | Macedonia | Karpasia | Simeto | Pisti | Mexikali81 | Korifla | Atlas | Anna | Duilio | Omrabi5 | Guard row |  |
|  |  |  |  |  |  |  |  |  |  |  |  |  |
| Guard row | Mesaoria | Karpasia | Duilio | Matt | Mexikali81 | Iride | Ourania | Simeto | Korifla | Hekabe | Guard row | Replication III |
|  |  |  |  |  |  |  |  |  |  |  |  |  |
| Guard row | Svevo | Pisti | Adnan2 | Claudio | Omrabi5 | Waha | Anna | Macedonia | Atlas | Aronas | Guard row |  |
|  |  |  |  |  |  |  |  |  |  |  |  |  |
| Guard row | Matt | Svevo | Waha | Aronas | Mexikali81 | Omrabi5 | Iride | Mesaoria | Ourania | Hekabe | Guard row | Replication II |
|  |  |  |  |  |  |  |  |  |  |  |  |  |
| Guard row | Anna | Atlas | Adnan2 | Duilio | Karpasia | Macedonia | Pisti | Claudio | Korifla | Simeto | Guard row |  |
|  |  |  |  |  |  |  |  |  |  |  |  |  |
| Guard row | Claudio | Matt | Adnan2 | Iride | Mexikali81 | Omrabi5 | Anna | Svevo | Waha | Duilio | Guard row | Replication I |
|  |  |  |  |  |  |  |  |  |  |  |  |  |
| Guard row | Macedonia | Korifla | Pisti | Ourania | Karpasia | Mesaoria | Atlas | Simeto | Hekabe | Aronas | Guard row |  |
|  |  |  |  |  |  |  |  |  |  |  |  |  |
| Guard row | | | | | | | | | | | |  |
