# Supplementary material for: Assessment of Vegetation Indices Derived by UAV Imagery for Durum Wheat Phenotyping under a Water Limited and Heat Stressed Mediterranean Environment
Source: Front Plant Sci. 2017 Jun 26;8:1114. doi: 10.3389/fpls.2017.01114 (PMC5483459; doi:10.3389/fpls.2017.01114)
Supplement: Supplementary file 2 [file Table_2.docx]

**Supplementary Table 2:** Pearson correlations between SVIs and photosynthetic pigments during the 1^st^ year

|  | **NDVI**  **booting** | **SR**  **booting** | **GNDVI**  **booting** | **NDVI**  **milk** | **SR**  **milk** | **GNDVI**  **milk** |
| --- | --- | --- | --- | --- | --- | --- |
| **SR booting** | 0.988** |  |  |  |  |  |
| **GNDVI booting** | 0.960** | 0.940** |  |  |  |  |
| **NDVI milk** | 0.498* | 0.458* | 0.555* |  |  |  |
| **SR milk** | 0.464* | ns | 0.524* | 0.966** |  |  |
| **GNDVI milk** | 0.680** | 0.673** | 0.689** | 0.875** | 0.853** |  |
| **Anthocyanin** | ns | ns | 0.445* | 0.827** | 0.809** | 0.709** |
| **Chlorophyll b** | 0.604** | 0.563** | 0.693** | 0.885** | 0.864** | 0.806** |
| **Chlorophyll a** | 0.573** | 0.528* | 0.655** | 0.871** | 0.848** | 0.780** |
| **Carotenoids** | 0.579** | 0.532* | 0.641** | 0.885** | 0.860** | 0.804** |
| **Total chlorophyll** | 0.577** | 0.533* | 0.660** | 0.873** | 0.851** | 0.784** |

ns (not significant), *p<0.05, **p<0.01
