# Supplementary material for: Assessment of Vegetation Indices Derived by UAV Imagery for Durum Wheat Phenotyping under a Water Limited and Heat Stressed Mediterranean Environment
Source: Front Plant Sci. 2017 Jun 26;8:1114. doi: 10.3389/fpls.2017.01114 (PMC5483459; doi:10.3389/fpls.2017.01114)
Supplement: Supplementary file 3 [file Table_3.docx]

**Supplementary Table 3:** Pearson correlations between SVIs, SPAD values and photosynthetic pigments during the 2^nd^ year

|  | **NDVI**  **heading** | **GNDVI**  **heading** | **SR**  **heading** | **SPAD**  **heading** | **NDVI**  **anthesis** | **GNDVI**  **anthesis** | **SR**  **anthesis** | **SPAD**  **anthesis** | **NDVI**  **milk** | **SR**  **milk** | **SPAD**  **milk** | **NDVI**  **dough** | **SR**  **dough** | **SPAD**  **dough** |
| --- | --- | --- | --- | --- | --- | --- | --- | --- | --- | --- | --- | --- | --- | --- |
| **GNDVI heading** | 0.873** |  |  |  |  |  |  |  |  |  |  |  |  |  |
| **SR heading** | 0.985** | 0883** |  |  |  |  |  |  |  |  |  |  |  |  |
| **SPAD heading** | ns | ns | ns |  |  |  |  |  |  |  |  |  |  |  |
| **NDVI anthesis** | 0.956** | 0.828** | 0.959** | ns |  |  |  |  |  |  |  |  |  |  |
| **GNDVI anthesis** | 0.958** | 0.889** | 0.963** | ns | 0.960** |  |  |  |  |  |  |  |  |  |
| **SR anthesis** | 0.921** | 0.801** | 0.949** | ns | 0.985** | 0.941** |  |  |  |  |  |  |  |  |
| **SPAD anthesis** | ns | ns | ns | 0.766** | ns | ns | ns |  |  |  |  |  |  |  |
| **NDVI milk** | 0.874** | 0.660** | 0.886** | ns | 0.947** | 0.894** | 0.942** | ns |  |  |  |  |  |  |
| **SR milk** | 0.853** | 0.634** | 0.875** | ns | 0.928** | 0.880** | 0.941** | ns | 0.989** |  |  |  |  |  |
| **SPAD milk** | 0.583** | 0.449* | 0.573** | 0.579** | 0.671** | 0.614** | 0.630** | ns | 0.710** | 0.658** |  |  |  |  |
| **NDVI dough** | 0.774** | 0.553* | 0.789** | ns | 0.870** | 0.803** | 0.874** | ns | 0.958** | 0.957** | 0.632** |  |  |  |
| **SR dough** | 0.763** | 0.535* | 0.781** | ns | 0.857** | 0.792** | 0.868** | ns | 0.952** | 0.960** | 0.632** | 0.998** |  |  |
| **SPAD dough** | 0.617** | ns | 0.633** | ns | 0.709** | 0.626** | 0.712** | ns | 0.806** | 0.777** | 0.845** | 0.805** | 0.798** |  |
| **Anthocyanin** | ns | ns | ns | ns | ns | ns | ns | 0.476* | ns | ns | 0.526* | 0.532* | 0.527* | 0.665** |
| **Chlorophyll b** | 0.533* | ns | 0.538* | 0.578** | 0.658** | 0.530* | 0.656** | ns | 0.752** | 0.722** | 0.853** | 0.767** | 0.757** | 0.931** |
| **Chlorophyll a** | 0.526* | ns | 0.544* | 0.528* | 0.649** | 0.539* | 0.658** | ns | 0.751** | 0.730** | 0.841** | 0.776** | 0.773** | 0.933** |
| **Carotenoids** | ns | ns | ns | 0.637** | 0.458* | ns | 0.452* | 0.476* | 0.550* | 0.508* | 0.757** | 0.584* | 0.569** | 0.813** |
| **Total chlorophyll** | 0.528* | ns | 0.544* | 0.535* | 0.651** | 0.538* | 0.659** | ns | 0.752** | 0.730** | 0.844** | 0.776** | 0.772** | 0.934** |

ns (not significant), *p<0.05, **p<0.01
