# Supplementary material for: Assessment of Vegetation Indices Derived by UAV Imagery for Durum Wheat Phenotyping under a Water Limited and Heat Stressed Mediterranean Environment
Source: Front Plant Sci. 2017 Jun 26;8:1114. doi: 10.3389/fpls.2017.01114 (PMC5483459; doi:10.3389/fpls.2017.01114)
Supplement: Supplementary file 4 [file Table_4.docx]

**Supplementary Table 4:** Pearson correlations of SVIs, SPAD values and photosynthetic pigments recorded at different years.

|  |  | **1^st^ year** | | | | | | | | | | |
| --- | --- | --- | --- | --- | --- | --- | --- | --- | --- | --- | --- | --- |
|  |  | **NDVI**  **booting** | **SR**  **booting** | **GNDVI**  **booting** | **NDVI**  **milk** | **SR**  **milk** | **GNDVI**  **milk** | **Anthocyanin** | **Chlorophyll b** | **Chlorophyll a** | **Carotenoids** | **Total chlorophyll** |
| **2^nd^ year** | **NDVI heading** | ns | ns | ns | 0.616** | 0.629** | 0.562** | ns | ns | ns | ns | ns |
|  | **GNDVI heading** | ns | ns | ns | 0.537* | 0.544** | 0.557* | ns | ns | ns | ns | ns |
|  | **SR heading** | ns | ns | ns | 0.614** | 0.623** | 0.559* | ns | 0.450* | 0.454* | 0.468* | 0.454* |
|  | **SPAD heading** | 0.511* | 0.520* | 0.505* | ns | ns | 0.579** | ns | 0.474* | 0.444* | ns | 0.448* |
|  | **NDVI anthesis** | ns | ns | ns | 0.619** | 0.635** | 0.576** | ns | ns | ns | ns | ns |
|  | **GNDVI anthesis** | ns | ns | ns | 0.560* | 0.563** | 0.561* | ns | ns | ns | ns | ns |
|  | **SR anthesis** | ns | ns | ns | 0.609** | 0.622** | 0.547* | ns | ns | 0.444* | 0.455* | ns |
|  | **SPAD anthesis** | 0.565** | 0.549* | 0.561* | ns | ns | ns | 0.466* | ns | ns | ns | ns |
|  | **NDVI milk** | ns | ns | ns | 0.637** | 0.650** | 0.565** | ns | 0.458* | 0.466* | 0.481* | 0.465* |
|  | **SR milk** | ns | ns | ns | 0.616** | 0.626** | 0.532* | ns | ns | 0.457* | 0.472* | 0.456* |
|  | **SPAD milk** | 0.559* | 0.577** | 0.549* | 0.625** | 0.613** | 0.736** | 0.534* | 0.656** | 0.641** | 0.651** | 0.644** |
|  | **NDVI dough** | ns | ns | ns | 0.606** | 0.624** | 0.491* | 0.498* | ns | 0.448* | 0.470* | 0.446* |
|  | **SR dough** | ns | ns | ns | 0.607** | 0.623** | 0.485* | 0.505* | ns | 0.455* | 0.476* | 0.453* |
|  | **SPAD dough** | ns | ns | ns | 0.722** | 0.720** | 0.635** | 0.599** | 0.711** | 0.722** | 0.703** | 0.721** |
|  | **Anthocyanin** | ns | ns | ns | 0.672** | 0.670** | 0.566** | 0.823** | 0.603** | 0.620** | 0.668** | 0.619** |
|  | **Chlorophyll b** | 0.489* | 0.476* | 0.499* | 0.766** | 0.769** | 0.735** | 0.685** | 0.740** | 0.728** | 0.727** | 0.730** |
|  | **Chlorophyll a** | 0.464* | 0.447* | 0.478* | 0.787** | 0.784** | 0.754** | 0.723** | 0.753** | 0.755** | 0.760** | 0.755** |
|  | **Carotenoids** | 0.488* | 0.454* | 0.495* | 0.677** | 0.666** | 0.720** | 0.636** | 0.616** | 0.603** | 0.618** | 0.605** |
|  | **Total chlorophyll** | 0.468* | 0.451* | 0.481* | 0.786** | 0.783** | 0.753** | 0.719** | 0.753** | 0.753** | 0.757** | 0.753** |

ns (not significant), *p<0.05, **p<0.01
